# Levofloxacin-Resistant *Haemophilus influenzae*, Taiwan, 2004–2010

**DOI:** 10.3201/eid2008.140341

**Published:** 2014-08

**Authors:** Shu-Chen Kuo, Pei-Chen Chen, Yih-Ru Shiau, Hui-Ying Wang, Jui-Fen Lai, Wen Huang, Tsai-Ling Yang Lauderdale

**Affiliations:** National Health Research Institutes, Zhunan, Taiwan (S.-C. Kuo, P.-C. Chen, Y.-R. Shiau, H.-Y. Wang, J.-F. Lai, I.-W. Huang, T.-L.Y. Lauderdale);; National Yang-Ming University School of Medicine, Taipei, Taiwan (S.-C. Kuo);; Taipei Veterans General Hospital, Taipei (S.-C. Kuo)

**Keywords:** Haemophilus influenzae, fluoroquinolone resistance, GyrA, ParC, Taiwan, respiratory infections, bacteria, antimicrobial resistance, levofloxacin

## Abstract

Levofloxacin resistance in *Haemophilus influenzae* has increased significantly in Taiwan, from 2.0% in 2004 to 24.3% in 2010 (p<0.001). Clinical and molecular investigations of 182 levofloxacin-resistant isolates revealed that the increase was mainly the result of the spread of several clones in the elderly population in different regions.

Since their first introduction in 1980s, fluoroquinolones have been used extensively (*1*). The wide use of these antimicrobial drugs has been attributed to the emergence of resistance in several bacterial species (*1*,*2*). Respiratory tract infection (RTI) is one of the most common conditions for which fluoroquinolones are used (*1*,*3*). *Haemophilus influenzae* is a major bacterial pathogen causing RTIs. Globally, fluoroquinolone resistance in *H. influenzae* has remained sporadic and uncommon, and international surveillance data showed the resistance rate to be <1% (*4**–**6*). However, emergence of fluoroquinolone-resistant *H. influenzae* strains has been reported in elderly patients and in those in long-term care facilities (*7**–**9*). 

The Taiwan Surveillance of Antimicrobial Resistance (TSAR) is a biennial nationwide surveillance program of inpatient and outpatient clinical isolates (*10*). Levofloxacin-resistant *H. influenzae* isolates were not detected in the TSAR collection before 2002 but emerged in 2004, and prevalence increased rapidly. We conducted a study to delineate the clinical and molecular characteristics of emerging levofloxacin-resistant *H. influenzae* in Taiwan.

## The Study

*H. influenzae* isolates were collected from 26 hospitals during 2004–2010 by the TSAR program, following previously described protocols (*10*). After species identification was confirmed, MICs were determined by reference broth microdilution method according to Clinical and Laboratory Standards Institute guidelines (*11*). Sensititre Standard HPB panels (ThermoFisher Scientific, East Grinstead, UK) were used. The MICs of levofloxacin, ciprofloxacin, moxifloxacin, gatifloxacin, and gemifloxacin of the levofloxacin-resistant isolates identified by broth microdilution were further determined by Etest (bioMérieux, Marcy l’Etoile, France). Pulsed-field gel electrophoresis (PFGE) and multilocus sequence typing (MLST) were performed on levofloxacin-resistant isolates following published protocols (*12*,*13*). Mutations in the quinolone resistance–determining regions (QRDR) of the drug targets *gyr*A and *par*C were determined by PCR and sequencing (*14*).

A total of 1,462 nonduplicate *H. influenzae* isolates were collected during the study period. Hospitals in the northern (32.1%), central (47.7%), and southern (18.7%) regions of Taiwan provided nearly all of the isolates. Specimens of respiratory origin (88.8%) comprised the largest proportion; few isolates (1.4%) came from blood specimens. Most (77.1%) patients were adults, and half (50.0%) were >65 years of age. 

Among the 16 antibacterial agents tested (Table 1), nonsusceptibility to levofloxacin, sparfloxacin, and trimethoprim/sulfamethoxazole increased steadily and significantly over the study period (p<0.05). Significant increases in levofloxacin and sparfloxacin resistance were the most prominent (p<0.001). The overall levofloxacin resistance rate increased from 2.0% (7/344) in 2004 to 10.6% (52/490) in 2006, 15.2% (49/323) in 2008, and 24.3% (74/305) in 2010 (p<0.001) (Table 1). In 2004, levofloxacin-resistant isolates were detected in 6 hospitals (2 in the south and 4 in the central region), but by 2010, isolates were detected in 19 hospitals in all 4 regions of Taiwan. The MIC_90_ of the 5 fluoroquinolones tested by Etest was >32 μg/mL for the 182 levofloxacin-resistant isolates detected by broth microdilution.

**Table 1:** Trends in antimicrobial nonsusceptibility in *Haemophilus influenzae* from the Taiwan Surveillance of Antimicrobial Resistance program, 2004–2010*

Antimicrobial agent	% Nonsusceptible					p value†	Odds ratio (95% CI)
	2004, n = 344	2006, n = 490	2008, n = 323	2010, n = 305	2004–2010, n = 1,462		
Amoxicillin/clavulanate	3.8	5.5	2.8	3.9	4.2	0.573	0.933 (0.731–1.189)
Ampicillin	61.3	56.5	49.2	59.3	56.6	0.242	0.943 (0.856–1.040)
Ampicillin/sulbactam	34.0	26.7	24.8	35.7	29.9	0.790	1.014 (0.913–1.127)
Cefaclor	54.4	48.2	53.6	57.0	52.7	0.241	1.060 (0.962–1.167)
Cefepime	2.6	1.0	0.0	2.0	1.4	0.287	0.790 (0.513–1.218)
Cefixime	4.4	1.8	0.6	2.3	2.3	0.044	0.698 (0.462–0.991)
Ceftriaxone	1.5	0.4	0.0	1.0	0.7	0.370	0.754 (0.406–1.398)
Cefuroxime	13.7	14.3	25.1	16.1	16.9	0.033	1.150 (1.011–1.307)
Chloramphenicol	39.8	37.8	28.8	33.1	35.3	0.01	0.875 (0.791–0.968)
Clarithromycin	40.7	50.4	58.5	43.6	48.5	1.148	1.704 (0.975–1.183)
Imipenem	3.8	3.3	1.9	3.0	3.0	0.333	0.867 (0.650–1.157)
Levofloxacin	2.0	10.6	15.2	24.3	12.5	<0.001	1.964 (1.675–2.302)
Meropenem	2.0	0.6	0.0	1.0	0.9	0.110	0.625 (0.351–1.112)
Sparfloxacin	4.9	15.1	19.5	26.9	16.1	<0.001	1.688 (1.472–1.936)
Tetracycline	40.7	38.6	30.7	33.4	36.3	0.010	0.875 (0.790–0.969)
TMP/SMX	67.4	66.5	71.8	74.1	69.5	0.023	1.131 (1.017–1.257)

*TMP/SMX, trimethoprim/sulfamethoxazole. †For the trend test calculation, a continuous variable was used as previously described (*10*).

Levofloxacin-resistant *H. influenzae* isolates were more commonly found in elderly patients, from respiratory origins, from regional hospitals and inpatient settings, and from central and southern Taiwan (Table 2). Multivariate analysis revealed that elderly patients (OR 3.601, 95% CI 2.435–5.325), regional hospitals (OR 2.054, 95% CI 1.379–3.059), geographic region (OR 3.656, 95% CI 2.214–6.038 for central; OR 5.428, 95% CI 3.050–9.611 for southern), and later study year (OR 2.13, 95% CI 1.692–2.395) were independent factors associated with levofloxacin resistance (Table 2).

**Table 2:** Factors associated with isolation of levofloxacin-resistant *Haemophilus influenzae*, Taiwan, 2004–2010

Factor	No. (%) isolates		p value*	Odds ratio (95% CI)	p value†
	Susceptible	Resistant			
Total	1,280 (87.5)	182 (12.5)			
Patient age >65 y	591 (80.8)	140 (19.2)	<0.001	3.601 (2.435–5.325)	<0.001
Respiratory tract specimen	1,123 (86.5)	175 (13.5)	<0.001		NS
Regional hospital	766 (85.0)	135 (15.0)	<0.001	2.054 (1.379–3.059)	<0.001
Inpatient hospital stay	849 (85.1)	149 (14.9)	<0.001		NS
Geographic region					
Northern	448 (95.5)	21 (4.5)	<0.001	Reference	
Central	585 (83.9)	112 (16.1)	<0.001	3.656 (2.214–6.038)	<0.001
Southern	225 (82.4)	48 (17.6)	0.006	5.428 (3.050–9.611)	<0.001
Eastern	22 (95.7)	1 (4.3)	0.346		
Study year				2.013 (1.692–2.395)	<0.001

*By χ^2^ test. †By multivariate logistic regression analysis. NS, not significant.

Among the 182 levofloxacin-resistant isolates, 153 could be grouped into 19 distinct clusters (A to S) (Figure). MLST was performed on 160 isolates, and a total of 50 sequence types (STs; ST630, ST787–ST802, ST1079–ST1095, and ST1097–ST1112) were identified. All but 1 ST, ST630, were new STs that had not been reported previously. However, most of the identified STs were single-locus variants (SLVs) of 7 ST types: ST788, ST790, ST792, ST793, ST795, ST799, and ST802. Some isolates of the same ST belonged to different PFGE clusters (Figure). PFGE and MLST results revealed emergence and regional predominance of some clones (Figure). For example, in the predominant STs (ST788 and its SLVs) in clusters A to F (n = 54, 29.7%), cluster A isolates were mostly from southern Taiwan and were found in all study years, whereas cluster B isolates were mostly from central Taiwan and found in later years (2008–2010). All but 1 of the isolates of ST799 and its SLV (n = 19) in clusters G–I were from central Taiwan, and most were from 2008–2010. Isolates of ST795 and its SLVs (n = 17) belonged to 3 clusters, were from central and northern Taiwan, and were recovered in later years (2008–2010).

**Figure:**
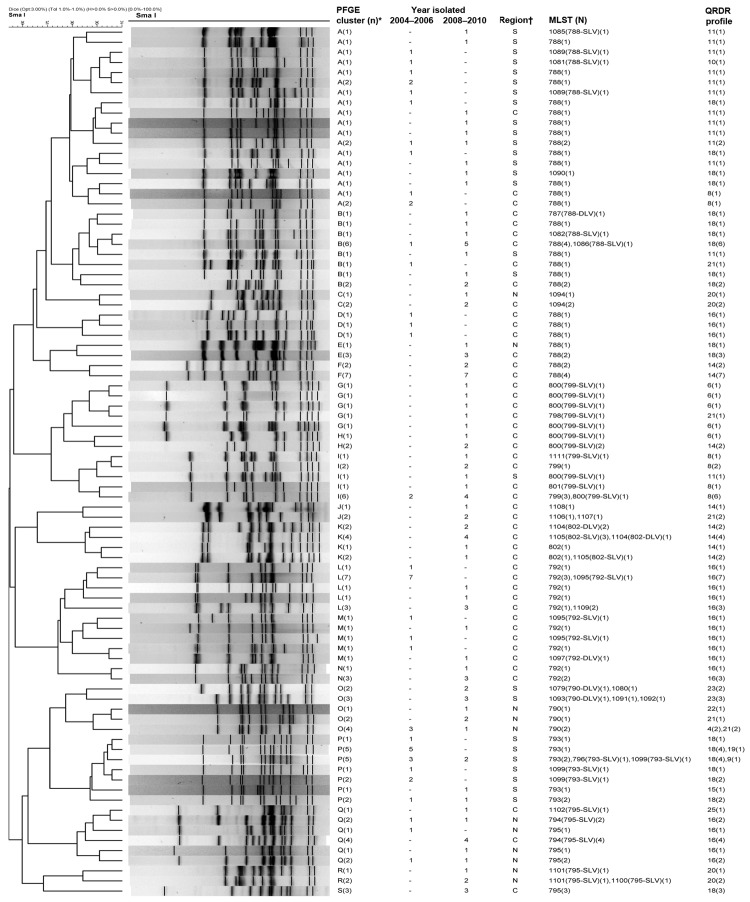
Dendrogram showing pulsed-field gel electrophoresis (PFGE) results for levofloxacin-resistant *Haemophilus influenzae* isolates digested by SmaI. *Salmonella enterica* serovar Braenderup strain H9812 (ATCC BAA664) was used as standard for DNA pattern normalization. PFGE patterns were analyzed by using BioNumerics software (Applied Maths NV, Sint-Martens-Latem, Belgium). For mutation profiles of the quinolone-resistance determining regions (QRDR) in GyrA and ParC, see Table 3. *Isolates having >80% similarity or <6 band differences were assigned a PFGE cluster if there were >3 isolates within the cluster; †region of hospital location: C, central; N, north; S, south. –, no isolates found. (n), number of isolates having the same PFGE pattern; MLST, multilocus sequence typing; (N), number of isolates on which MLST was performed; SLV, single locus variant, DLV, double locus variant.

All levofloxacin-resistant *H. influenzae* isolates had >2 mutations in the QRDR of *gyr*A and *par*C. A total of 25 QRDR mutation patterns were found (Table 3). The amino acid changes at residues 84 and 88 in GyrA and ParC that we found are the same as those found in previous reports (*1*,*14*,*15*). Most isolates of the same PFGE cluster had similar QRDR mutation patterns. However, isolates within the same PFGE cluster that had the same ST or its SLV but different QRDR mutation patterns were also found (Figure).

**Table 3:** Amino acid changes in GyrA and ParC quinolone resistance-determining regions of levofloxacin-resistant *Haemophilus influenzae* isolates, Taiwan, 2004–2010*

Mutation profile no.	Change in GyrA			Change in ParC		No. isolates
	S84	D88		S84	E88	
2	–	N		I	–	1
3	A	Y		I	–	1
4	D	Y		I	–	2
5	F	–		I	–	1
6	F	G		I	–	5
7	F	G		R	–	1
8	F	N		I	–	14
9	F	N		R	–	2
10	F	Y		–	K	1
11	F	Y		I	K	16
13	L	–		R	K	1
14	L	G		I	–	23
15	L	G		I	K	1
16	L	G		R	–	41
17	L	N		–	–	2
18	L	N		I	–	42
19	L	N		R	–	2
20	L	Y		–	K	7
21	L	Y		I	–	8
22	L	Y		I	K	1
23	V	N		I	–	7
24	Y	H		–	K	1
25	Y	N		I	–	1
26	Y	N		I	K	1

*PCR products were sequenced and sequences aligned by using Vector NTI (Invitrogen, Carlsbad, CA, USA). Amino acid codes: A, alanine; R, arginine; N, asparagine; D, aspartate; E, glutamate; G, glycine; H, histidine; I, isoleucine; L, leucine; K, lysine; F, phenylalanine; S, serine; Y, tyrosine; V, valine. –, no change.

## Conclusions

Our study indicates that levofloxacin-resistant *H. influenzae* emerged in Taiwan around 2004 and increased over the next 6 years, especially in elderly patients, regional hospitals, and central and southern Taiwan. We found age and regional differences in this resistance phenotype, which might have resulted from differences in fluoroquinolone use and patient populations. 

Longitudinal and international surveillance data from North America, Europe, and other regions have found low fluoroquinolone resistance in *H. influenzae* (<1%) (*4**–**6*). However, emergence and spread of fluoroquinolone-resistant *H. influenzae* have been reported. In 2001, fluoroquinolone-resistant *H. influenzae* spread in a hospital-affiliated long-term care facility in New York, NY, USA, and almost all resistant isolates belonged to 1 clone (*8*). From 2002 to 2004, the rate of fluoroquinolone resistance in *H. influenzae* in hospitals in the Hokkaido Prefecture in Japan increased from 0.5% to 2.6%, and the resistant isolates were found only in elderly patients (*9*). In 2007, a high rate of levofloxacin resistance (41.7%, 20/48 isolates) was found in *H. influenzae* that was colonizing 4 nursing home residents in southern Taiwan, and 2 major clones accounted for 90% (18/20) of the resistant isolates (*7*).

In our study, isolates of the same PFGE cluster mostly had similar mutation patterns. Therefore, the emergence of fluoroquinolone-resistant isolates may be the results of several clones spreading in the same region. However, isolates within the same PFGE cluster having the same ST but different QRDR mutation patterns were also found. In addition, nearly all the levofloxacin-resistant *H. influenzae* isolates belong to 7 previously unreported STs or their SLVs. This finding indicates that the emergence of levofloxacin-resistant isolates likely occurred through spontaneous mutation of hypermutable clones under selective pressure (*8*,*14*), and these clones then disseminated locally in each region.

In summary, our study revealed the emergence and spread of levofloxacin-resistant *H. influenzae* in different regions of Taiwan, with regional predominance of certain STs. We studied a large number of levofloxacin-resistant *H. influenzae* from clinical diagnostic samples of multiple hospitals in different regions of Taiwan, so physicians should take into account the high rate of fluoroquinolone resistance when they prescribe empirical therapy for *H. influenzae* infections. Surveillance and intervention measures should be directed at the risk groups identified in this study to halt the increase in fluoroquinolone resistance in *H. influenzae*.
